# Legacy lessons from the COVID-19 era to improve trial participation and retention: Views from trial participants, PPIE contributors and trial staff across the NIHR portfolio

**DOI:** 10.1371/journal.pone.0296343

**Published:** 2024-02-21

**Authors:** Heather Leggett, Karen Vinall-Collier, Julia Csikar, Sophy Barber, Rachel Carr, Amrit Bhatti, Sue Pavitt

**Affiliations:** 1 York Trials Unit, The University of York, York, United Kingdom; 2 School of Dentistry, The University of Leeds, Leeds, United Kingdom; University of Pretoria, SOUTH AFRICA

## Abstract

**Background:**

The Covid-19 pandemic had a profound effect on the delivery of healthcare research. Covid-19 research was prioritised and many non-essential trials were paused. This study explores the engagement experiences of trial participants’, PPIE contributors’ and trial staff during the Covid-19 pandemic and towards recovery and restoring a diverse and balanced UK clinical trials portfolio.

**Methods:**

Interviews and focus groups were undertaken with PPIE contributors, trial participants and trial staff members from NIHR research trials across the UK (November 2020-June 2021) across portfolio specialities: Cancer, Oral and Dental Health, Musculoskeletal Disorders, Cardiovascular Disease, Neurological Disorders, Primary Care, and Conditions associated with susceptibility to Covid-19 (Diabetes, Stroke, Respiratory Disorders). Topic guides were developed for each participant group and interviews were conducted over Zoom. The transcripts were analysed using codebook thematic analysis in NVivo (V.12).

**Results:**

106 participants comprising, 45 PPIE contributors, 27 trial participants and 34 trial staff members were recruited. Three themes to engagement with trials during Covid-19 were developed. *1) Ensuring continued contact*. Continued and tailored communication, having a trial point of contact and regular updates all enhanced trial engagement and retention. Patients’ unfamiliarity with materials being sent electronically reduced engagement and trust. *2) A balanced move to remote consultations*. Remote follow-up and monitoring were convenient and allowed for wider recruitment across the UK. Participants were more likely to discuss personal subjects in their own homes. Remote visits lacked a personal touch, some concerns over missed diagnoses or being unable to appreciate the situation, technical abilities or equipment failures were seen as barriers, especially for disadvantaged or older people. *3) The importance of feeling fully informed*. Factors that supported attendance were knowledge about trial conduct adherence to Covid-19 regulations, social distancing, clear signage at the site and opportunities to ask questions. Barriers included not knowing what to expect and not feeling safe with rules and regulations.

**Conclusions:**

Our findings highlight a number of ways to future proof trial delivery against future pandemics or disruptions such as offering online options to participate in research, ensuring consistent communication between participants and the research team, making sure participants feel fully informed and the continued reassurance of safety in the clinical setting.

## Introduction

The emergence of the Covid-19 pandemic in early 2020 sent a shockwave through life as we knew it. Within the United Kingdom, the National Health Service (NHS) restructured its delivery of care by redeploying all non-urgent care staff to frontline Covid-19 services [1]. The National Institute for Health Research (NIHR) were called upon to prioritise Covid-19 research, pause existing non-essential trials, and clinical staff were deployed to Covid-19 activities. A small proportion of non-Covid-19 studies remained open, largely to maintain safety in follow-up.

The Government introduced a directive, ‘Stay home, Stay Safe, Protect the NHS’ which introduced social distancing, working from home, wearing face masks in public places and ‘shielding’ for vulnerable groups to reduce the spread of Covid-19 [2]. Compliance was supported through a combination of legislation such as the Coronavirus Act 2020 and public health messaging. Adherence with safety measures were ‘almost always’ followed by the population [3].

In May 2020, the NIHR published its ‘Framework for restart’[4] which outlined the restoration of the non-Covid-19 portfolio of research they fund and support. The remobilisation of non-Covid research was seen as critical to advancing scientific knowledge and continuing innovation in treatment. It has been reported that participation in clinical research has declined since the onset of the pandemic [5]. Sheridan (2020) and colleagues noted that research participation, even before the onset of Covid-19, had a low uptake [6]. They cited the low uptake stemming from a lack of knowledge on the purpose/function of research and if the research was safe to participate in. Other concerns included: feeling that their privacy or confidentiality may be breached, being used as a ‘guinea pig’, and a general mistrust of researchers’ intentions [6]. This hesitancy to participate in research has been compounded, especially if in-person visits to a clinical setting were required [7] Concerns during the pandemic included: [1] difficulties attending trial sessions due to reduced public transport or not wanting to use public transport, [8] difficulties understanding safety protocols or perceptions that health sites were not stringent enough and [2] worry that visiting health settings could pose a threat to the participant’s health from infection of Covid-19 [6,9]. Studies operating during the paused phase struggled to recruit and retain study participants [1].

There is now an opportunity to learn from what we have been through during this global pandemic to future-proof research participation. Gathering data from participants, patients, and staff delivering research trials will help us to understand what can support confidence and promote a desire to participate in research. What were the key drivers that maintained research activity, what worked, what was less successful and how can we build this into our delivery of research so that these vital lessons from all stakeholders are not lost?

The aim of this research was to explore trial participants’, patient and public involvement and engagement (PPIE) contributors’ and trial staff members’ experiences of engagement with trials during Covid-19 across different specialty areas and towards recovery and restoring a diverse and balanced UK clinical trials portfolio.

## Methods

### Study design

Semi-structured interviews and focus groups were undertaken with PPIE contributors, trial participants and trial staff members on NIHR funded projects or NIHR Clinical Research Network (CRN) portfolio studies across the UK. Ethical approval was granted from the Dental Research Ethics Committee 250820/JC/307 to for the PPIE contributors and HRA approval (REC: 20/PR/0633) for the trial participants and trial staff members (S1–S3 Files). The methods are reported in line with the COREQ checklist (S4 File)

### Patient, Public Involvement and Engagement (PPIE)

PPIE played a key role in the design and objectives, and drove the rationale, with four members of the research team being PPIE group co-ordinators. The UK standards for Public Involvement (nihr.ac.uk) were upheld at the forefront of our design and approach. PPIE members of the University of Leeds School of Dentistry group (SMILEAIDER) had involvement throughout the research, from early discussions around conceptualisation of the project and assessing its burden, to commenting on and developing patient information sheets and topic guides for interviews/focus groups, as well advising on how to approach trial participants and PPIE contributors during recruitment. They were regularly consulted throughout the study, and we have also taken our findings to them to discuss our interpretation. The study also involved participation with PPIE groups from 8 different specialities invited to take part in the research for their role as PPIE members. The study was also promoted through the NIHR hosted website—Be Part of Research—to maximise spread across the UK. Dissemination has utilised the PPIE groups that were involved in the interviews, to widen our reach to members of the public.

### Participants and recruitment

**Fig 1:**
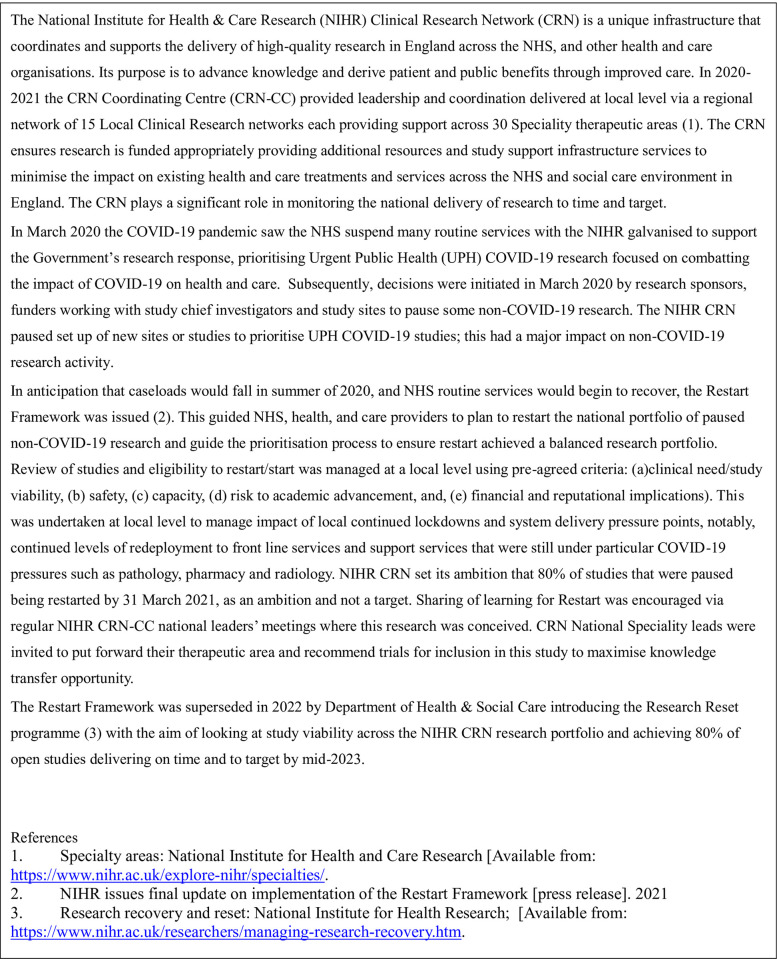
The NIHR clinical research network and UK research infrastructure and its’ response to restarting the NIHR portfolio of studies during COVID-19.

NIHR Clinical Research Network CRN National Speciality leads across the 30 speciality areas (Fig 1) were invited to put forward their therapeutic area and recommend trials for inclusion in this study to maximise knowledge transfer opportunity and gain understanding about study adaptation for trial participant recruitment and retention to inform NIHR Restart and future efficient, acceptable trial delivery considerations (Fig 1). This led to a broad sampling framework spanning nine NIHR specialist therapeutic areas with a range of acute and chronic conditions, with participants spanning secondary care/tertiary care, primary care and community non-NHS settings with varied shielding recommendations. Specifically, the therapeutic areas were: Cancer; Cardiovascular Disease; Musculoskeletal Disorders (e.g. Rheumatoid arthritis), Neurological Disorders (e.g. Multiple Sclerosis & Parkinson’s); Oral and Dental Health (including non-NHS settings, such as studies in educational settings); Primary Care; and conditions associated with health inequalities that early in the pandemic were suspected to have susceptibility/ poorer outcomes if they contracted Covid-19 (e.g. Diabetes, Stroke, Respiratory Disorders (e.g. COPD)). At least two trials in each speciality area agreed to participate in the study. We did not purposefully sample by trial design complexity; sampling was participant centred not focussing on trial delivery mechanism. We used convenience sampling to recruit all participants. In keeping with current methodological guidance, we aimed for maximum variation in our sample, rather than saturation [10]. We aimed for maximum variation according to age, gender and ethnicity for members of PPIE groups and trial participants.

For each trial, participants were recruited from three groups:

1. Members of PPIE groups (related to the participating trials for each condition where possible)
2. Trial participants
3. Trial staff members

Participants were recruited in the following ways:

1. PPIE contributors from each trial (PPIE) were contacted through the trial principal investigator or their delegate and invited to participate in a qualitative study. Where we struggled to identify or recruit participants from a particular speciality, PPIE contributors were recruited through the online platform People in Research (www.peopleinresearch.org). These contributors were recruited through an online advert for individuals who were currently involved in PPIE work in that speciality area.
2. Trial participants (TP) were approached via the principal investigator for each trial or their delegate from the clinical trial team and invited to take part in a qualitative study. Participants were approached via the post, email, text or telephone, depending on their usual communication preference. A reminder was typically sent 2–4 weeks later.
3. Trial staff members (TSM) were identified through contact with the trial principal investigator or their delegate and invited to participate in a qualitative study.

For all three groups, interested participants were asked to directly contact the research team. They were then sent a participant information sheet and asked to complete a consent form and return it to the research team. Trial participants and PPIE contributors were also asked to complete a demographics form. All identifiable participant information was stored in a password protected file on password protected computers.

### Data collection

For PPIE contributors and trial participants, focus groups (organised by speciality and trial) were arranged at a time that most suited participants. If necessary, additional one-to-one interviews were also undertaken. Trial participant and PPIE contributors were offered a £20 online voucher for participating in an interview/focus group. For the trial staff members, one-to-one interviews were undertaken at a time that suited them. In some instances, staff who worked on the same trial were interviewed together at their request. Separate focus groups were undertaken for each participant group and each speciality. Across all participant groups, all interviews and focus groups took place over Zoom and were recorded. Interviews and focus groups were undertaken by several different researchers (HL, KVC, JC, AB, RC, ZM, JA, RB, JCo, JT). All interviews and focus groups were undertaken by two researchers: an experienced qualitative researcher who led the interviews/focus groups and a junior researcher who monitored the chat functions on Zoom and acted as a facilitator. Interviewers were not known to or involved in the trial of the trial participants and PPIE contributors they interviewed nor did not work with any of the trial staff members they interviewed.

A topic guide was used to guide the conversation in all interviews and focus groups (S5 File). Topic guides were developed for each group of participant types to ensure the questions and focus was specific to their perspective and experiences of trial involvement during the pandemic. The topic guides were developed by the research team in consultation with our PPIE group and literature surrounding Covid-19 and trials.

### Analysis

All interviews were transcribed, and the transcripts imported into NVivo (v12) to assist with data management during analysis. We used codebook thematic analysis to analyse the interviews and focus groups [11]. PPIE contributor focus groups and interviews were undertaken first, so analysis began with these transcripts before the completion of the trial participants and trial staff member focus groups/interviews. The PPIE contributors’ transcripts were analysed inductively to search for broad topics/areas relating to how participants felt about returning to research or hospital settings during Covid-19 and what would make them feel safe during this time. Double coding of 4 transcripts by HL, KVC, AB and RMC, followed by group meetings to discuss code and theme development led to the formation of a coding framework which was then used by HL to code the remaining PPIE contributor focus groups/ interviews and the trial participant focus groups/interviews in a more deductive manner. Trial staff member interviews were coded by HL and were discussed with KVC using the same coding framework but also inductively to elicit issues that related only to staff. Any concerns or issues over coding were reviewed between HL and KVC and resolved through discussion. After initial coding, HL and KVC worked together to refine and bring together the themes from the trial participants, PPIE contributors and the trial staff members to reflect barriers and facilitators to engagement in trials.

## Results

**Fig 2:**
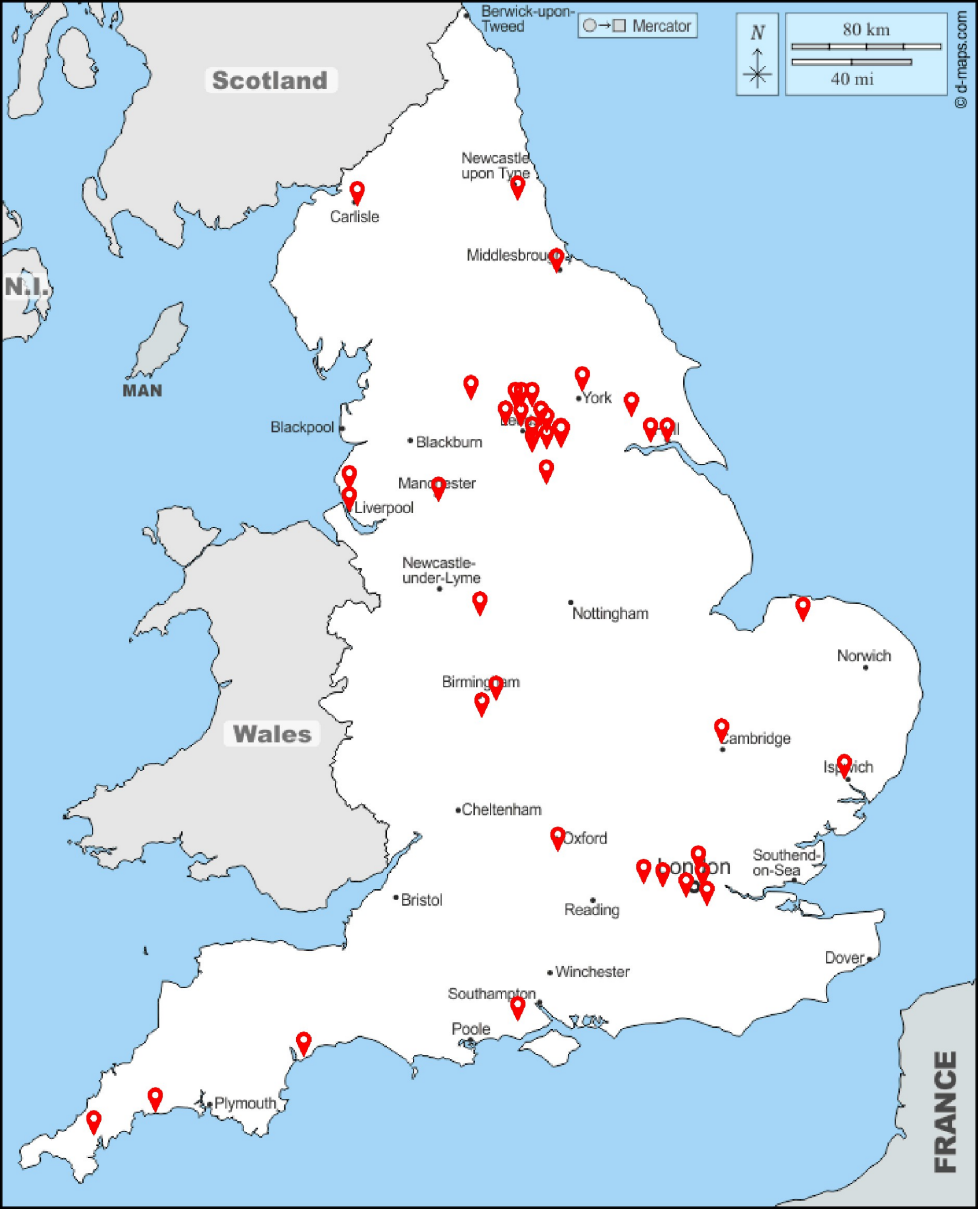
Geographical distribution of trial participants and PPIE contributors.

Interviews and focus groups took place between November 2020 and July 2021 with PPIE contributors and between January 2021 and July 2021 with trial participants and trial staff members. Participants were typically providing their perspective of involvement in research during the entire pandemic period preceding their interview date. A summary of the Covid-19 pandemic timeline of the UK government coronavirus lockdowns and measures, March 2020 to December 2021 has been summarised by the Institute of Government Analytics. This provides a useful historical context of the key pandemic milestones in the UK [12] and how they intersect with this study sample period. A total of 11 focus groups and 23 individual interviews were conducted during this period. The focus groups consisted of 2–7 participants for PPIE contributors, 2–5 participants for trial staff members, and 5 participants for trial participants. Interviews lasted between 30 minutes and 60 minutes and focus groups lasted between 60 and 90 minutes. In total, there were 106 participants consisting of 45 PPIE contributors, 27 trial participants and 34 trial staff members. This is shown per speciality in Table 1. Trial participants and PPIE contributors were recruited from across the UK, which maximised geographical variation and enabled us to capture experiences of Covid-19 and trials from across the UK, as shown in Fig 2.

**Table 1:** The number of participants per speciality and participant group, including details of focus groups and interviews conducted.

Specialty area	PPIE contributors (PPIE)	Trial participants (TP)	Trial staff members (TSM)	Total
**Cancer**	**5** *(1 focus group n = 5)*	**3** *(interviews n = 3)*	**5** *(1 focus group n = 4*, *interview n = 1)*	13
**Oral and Dental Health**	**5** *(1 focus group n = 5)*	**1** *(interview n = 1)*	**7** *(1 focus group n = 2*, *interviews n = 5)*	13
**Musculoskeletal Disorders **	**6** *(1 focus group n = 6)*	**5** *(interviews n = 5)*	**4** *(interviews n = 4)*	15
**Cardiovascular**	**7** *(1 focus group n = 7)*	**2** *(interviews n = 2)*	**2** *(interviews n = 2)*	11
**Neurological Disorders **	**11** (*1 focus group n = 7*, *interviews n = 4)*	**9** *(1 focus group n = 4*, *interviews n = 5)*	**5** *(interviews n = 5)*	25
**Primary Care**	**2** *(1 focus group n = 2)*	**1** *(Interview n = 1)*	**7** *(1 focus group n = 5*, *interviews n = 2)*	10
**Diabetes, Stroke, Respiratory Disorders**	**9** *(1 focus group n = 8*, *interviews n = 1)*	**6** *(Interview n = 6)*	**4** *(Interview n = 4)*	19
**Total**	45	**27**	**34**	106

Diabetes, Stroke, Respiratory Disorders were a group of conditions associated with health inequalities that early in the pandemic were suspected to have susceptibility/ poorer outcomes if people with these conditions contracted Covid-19.

Thematic analysis generated three themes that described barriers and facilitators to engagement with trials during Covid-19: 1) ensuring continued contact, 2) a balanced move to remote consultations and 3) the importance of feeling fully informed. The results reflect trial participants’, PPIE contributors’ and trial staff members’ perspectives on patient engagement. Quotes from the participants use the following ID: *“Speciality area code_Participant type_Interview number or Focus group member and participant number” (e*.*g*., Cardiovascular Disease_trial staff member_INT01*)*.

### Theme 1: Ensuring continued contact

Ensuring continued contact was key to trial participant and PPIE engagement when faced with a reduction or complete halt in face-to-face visits. Trial staff feared a large impact on the retention of participants as well as recruitment of new participants. In reality, it was seen to be easier to retain existing participants in the trial but harder to recruit new participants. Recruitment and engagement were also influenced by the prevalence of current Covid-19 cases and the reporting of Covid-19 in the news: *“So it does go a bit up and down as the news*, *you know*, *people get more worried and then settle down a bit and then…” [Cardiovascular Disease_Trial staff member_INT01]*.

There was great variation across different trial specialities regarding the cessation of face-to-face contact. Some trials, such as those in Oral and Dental Health and Diabetes, were still paused at the time of the interview in 2021, whereas within Cancer trials, those that were deemed essential to care did not pause, but instead, accommodations were made to ensure delivery could continue.

Cancer_Trial staff member_INT01: “If the patients were desperately unhappy to come in we were completely understanding about that. But generally, you know, with the condition that we were dealing with I think that’s the key thing. This isn’t a condition you could just say, ‘come back in a year and everything’ll be fine’. We’ve seen the consequences of trying to do that is that we’ve got a big problem with cancer. So and the patients very quickly were more fearful of substandard care and surveillance than they were of Covid. Because of its cancer and, and……the leverage that that creates in the patient’s mind.”

Contact was usually achieved through telephone calls, emails, texts, and postal letters; of these, telephone communication was seen as a key facilitator to trial participant and PPIE engagement. During the Covid-19 pandemic, it became even more important that these methods of communication were tailored to the demographics of the group they were trying to reach. This included using simple English language, providing materials in other languages or in different formats (such as infographics and large text) and asking people’s preferences for how they wanted to be contacted (phone, email, post), More elderly patients and PPIE contributors preferred to be contacted via telephone or post rather than email. From the trial participants’ and PPIE contributors’ perspective, it was clearly important that plain language was used and there were preferences for how information was presented: to have the most important information first, use different font sizes, avoid capitals and exclamation marks and set a clear date or action required. Participants who received regular updates about the trial, and their involvement in it, felt more informed about the running of the trial, they remained more engaged with it and were more likely to feel comfortable attending clinical settings for appointments. In contrast, not being able to get in touch with anyone from the trial was a worry for patients when the trials were paused. These patients also felt left in the dark regarding whether the trial was continuing or not in the long-term.

Diabetes, Stroke and Respiratory Disorders_PPIE_FG01_P07: “But the guys who write thinking that they’re writing plain English clearly haven’t had a conversation in their life with a real human being. So, you know, the quality, the quality of written communication coming from the NHS is appalling.”

Neurological Disorders _Trial participant_INT03: “Yeah, I mean, yeah, the team are very clear from the beginning what it is, what it isn’t, what your kind of part of the trial is, and I think they’re also very good at keeping in touch with people as you go along.”

Primary Care _Trial participant_INT01: “I think in general it comes down to two things which is that information has to be comprehensive and very clear they’re not littered with medical jargon and I think the participant has to have a very clear idea of exactly what’s going to happen at what stage, and you know set it all out and communication is key.”

Although this was not reported by trial participants, trial staff reported problems when encouraging trial participants to engage with electronic materials -they found that participants were less likely to return questionnaires or other forms via email compared to the completion of paper-based materials in the clinical setting. Staff also felt that they spent a lot of time chasing people and had increased workloads as a result.

Oral and Dental Health _Trial staff member_INT03: “Spending a lot of time explaining procedures in the hospital, reassuring that coming to the hospital was safe and just really trying to put patients at ease that being part of the trial was safe and that we were taking every step possible to make sure that, that they weren’t compromising them by keeping them in the trial…lot more co-ordination…know what drugs their on…. and it was actually safer for them to continue in the trial rather than just taking them off of a, off of a drug. It probably increased my workload per patient.”

Neurological Disorders _Trial staff member_INT01: “So there is, there’s a lot of chasing and it’s, it’s, it can be quite heavy, we do a lot of patient reported outcomes. So that’s sort of thirty five pages of questionnaires that we need to do over the phone so it’s…some a’ the patients, they can get a bit fed up.”

Trial participants having a main point of contact within the trial gave them reassurance that any issues or questions they had would be resolved. Those who had a reliable point of contact reported feeling much more ‘kept in the loop’ regarding the progression of the trial, their involvement and medication, and any Covid-19 related information. As such, many trial participants and PPIE contributors had a great deal of trust in their clinical team and their decisions around when it was safe for them to visit clinical settings. Patients felt that their physicians would not let them visit hospital if it was not a safe environment for them.

Cancer_Trial participant_INT01: “I thought about it logically and, you know, and thought if it wasn’t safe to go in, them knowing my situation, they wouldn’t have sent for me.”

Neurological Disorders_Trial staff member_INT01: “She [research nurse] did a lot of zoom sort of coffee mornings with the patients to get their feedback and that was where a lot of them said they really appreciated the contact and being able to know that a service was still running.”

Neurological Disorders_Trial staff member_INT02: “Two members of our team, they have monthly zoom meetings with our patients. So the patients all, can all talk to each other and……see how, how they’re going so they’re all in the same boat.”

### Theme 2. A balanced move to remote consultations

It was common for consultations or follow-up appointments to be held online or via telephone. Video conferencing was viewed very favourably as a remote alternative for research appointments, mainly due to its convenience. Trial staff members found the transition to remote visits relatively quick and easy; they appreciated that it allowed them to keep up with their participants as well as allow for wider recruitment from across the UK rather than regional. Remote visits were seen as more efficient since *"there’s no way you’d turn somebody round from a face-to-face visit in ten to fifteen minutes*, *whereas it’s perfectly acceptable to do that on a Zoom” [Neurological Disorders _Trial staff member_INT03]*. Another benefit to staff and trial participants was patients being more comfortable in their own homes and it being “quieter”. Consequently, this made them feel more relaxed and were more likely to discuss personal subjects with their care team and subsequently were more engaged with the trial.

Diabetes, Stroke and Respiratory Disorders _Trial participant_INT02_P02: “It was convenient that I could just do it. I didn’t have to travel anywhere or anything like that, worry about parking or any kinda that sorta side a’ things. And I guess with what was going on out there with the pandemic it, it was, it gave me that reassurance that I’m still cocooned in my home and safe.”

Cancer _PPIE_FG01_P04: “I’m on the wrong end of the age scale for computers, but I’ve gotten more used to it. I’ve been on quite a few of these zoom meetings, different people at [hospital name]… and I’m much more relaxed and I get, oh I don’t know… I’m just, I’m more comfortable with them than I used to be.”

Despite the benefits afforded by remote consultations, trial participants and staff did experience limitations with the transition. Amongst all participant groups there was a pervasive feeling that *“It’s the best alternative but it doesn’t go quite the same*… .*conversation doesn’t flow in the same sort of way*…*elements of the conversations can be lost*. *And also*, *you can’t have the same kind of interaction that you can if you’re sitting with a group of people” [*Cancer *_PPIE_FG01_P03]*. Furthermore, trial participants and PPIE contributors often felt that a remote consultation was not appropriate for a diagnosis as the doctor could not properly see or feel any important symptoms. It was believed that trial participants could suffer because of this by missing important diagnoses. Additionally, difficulties accessing the required technology due to participant’s technical abilities or issues with the equipment (computers or internet not working) acted as barriers to remote consultations. It was felt that these disadvantaged older people in particular.

Musculoskeletal Disorders_PPIE_FG01_P01: “I know from my own experience that when I talk to the doctors on the phone, not even on zoom that unless you actually very precisely describe what your symptoms are then they can get a completely different picture of the diagnosis from if they actually saw you. And not many people can do that, you’ve got to be a bit in the profession to be able to explain to the doctor in so many words exactly what’s wrong and your needs. Especially if its anything with your inner organs. It can never take the place of face-to-face appointments and someone will suffer in the process.”

Trial staff also recognised this limitation and felt that “*remote’s fine but there’s only so much you can capture… It is very subjective to what the patient reports*. *We can’t sort of observe so there is limited data–that’s one thing we found difficult” [Neurological Disorders_Trial staff member_INT01_P01]*. Remote appointments also made it more difficult (and sometimes impossible) to undertake assessments that required examination, close observation or specific medical equipment. The move online also limited trial staffs’ informal contact with carers/relatives of trial participants. This limited the practitioner’s ability to fully explore the participant’s well-being and any side effects through informal conversations with their carers/relatives. This was also true when carers were not allowed into face-to-face visits.

Neurological Disorders_Trial staff member_INT03: “It then just feels very artificial saying, ‘actually I really wanted to speak to your care partner also’……’because I can’t just take your word for it….”

Trial staff described how the success of remote consultations had led to unforeseen consequences of participants preferring remote visits; *“a lot of them are reluctant to come back in for face-to-face visits now*.. *they really prefer the remote visits… but there’s only so much we can capture” [Neurological Disorders*_*Trial staff member_INT01]*. This appeared to mostly be those who were more likely to need more encouragement and engagement to come into the hospital before the pandemic, *“the patients that were a bit on the edge anyway and needed a lot of engagement to keep them coming*, *those ones have been very difficult to maintain” [Neurological Disorders __Trial staff member_ INT01]*. Whilst efficiencies were seen when many participants and routine visits moved to remote delivery, trial staff workloads were significantly increased in terms of maintaining some participants. Another form of remote delivery that occurred during this time was a move to home visits. Staff found that a lot of work was involved in arranging home visits as it often involved liaising with home phlebotomy, pharmacy delivery and general practice -*“so we did quite a lot to keep them in the trial and keep the trial going” [Neurological Disorders _ Trial Staff Member _INT01]*. *Capturing* all the participant safety data for the trial was a concern and led to prioritisation of solutions for remote and “safe deviations” from the protocol.

A move towards remote trial delivery meant that trial participants and PPIE contributors lost the social contact and peer support that comes from face-to-face trial delivery. It was felt that the running of online peer support groups (separate to any planned meetings or consultations) had a positive impact on trial participants’ and PPIE contributors’ engagement and sense of community. From a staff perspective, remote working was seen to reduce cohesion and engagement as a trial team and with trial participants and PPIE contributors.

Neurological Disorders_Trial staff member_INT01: “And the remote working you do really lose that team spirit I think don’t you. So it’s hard to push on but yeah we’re doing okay generally.”

Neurological Disorders_Trial participant_FG01_P02: “Thing is, perhaps it might not have happened if it hadn’t been for Covid. There’s a group of us who are old patients who’ve sort of found each other by going to clinics and information from just localised contexts, but they range from Scotland down to Cornwall, and we meet on a Zoom meeting once a week… And we can actually chat about the problems that we’d have, but you also get to know the people themselves. It’s very laidback, but very supportive, cause when someone has a problem, someone of our group, so probably, cause of how long we’ve been at it, know something”

### Theme 3. The importance of feeling safe and informed of new rules

Knowing what to expect when returning for face-to face clinical visits and feeling safe in those settings facilitated trial participants’ engagement with the visit. If they were to return to clinic visits, many PPIE contributors and trial participants spoke about wanting to know about the cleanliness and space to comply with social distancing recommendations before visiting either via a leaflet, video, or talking to someone over the telephone about the visit. The opportunity to ask questions and to know that it was a space that was sufficiently clean and could accommodate social distancing from one another was important. Those who continued to go into hospital talked positively about how *“it’s been very carefully managed” [Diabetes*, *Stroke*, *Respiratory Disorders_Trial participant_INT01_P01]*. Recounting being the only person in the waiting room and being ushered in to be seen as soon as they arrived aided trial participants’ confidence. Alternative entrances to sites (not the main entrance) or other assurances of non-contact with people with Covid-19 was also appreciated.

Neurological Disorders_PPIE_INT04: “It’s [research visit] a risk which I don’t have to take. Yeah so, you know, if, if a hospital can be avoided I would appreciate that….. because I don’t want to expose myself to risk, to unnecessary risk”.

Knowing and understanding the Covid-19 regulations that would be in place in advance of attending a clinical setting helped participants feel confident in their safety. It was important that the information was comprehensive and transparent so that participants and PPIE contributors fully understood it and knew what was expected of them. It was also important for this feeling of safety to be reinforced during their visit to the hospital through clear signs, one-way systems and staff members providing instruction. These regulations included but were not limited to: other patients wearing face masks, social distancing being facilitated and adhered to, staff wearing full Personal Protective Equipment (PPE), there being no unnecessary proximity with staff during treatment, one way systems inside the hospital to regulate the flow of people, the department not being too crowded, shorter waiting times, Perspex screens being in place, enhanced cleaning, avoiding acute wards, not being near Covid-19 patients, good ventilation and easy access to hand sanitiser.

Cardiovascualar Disease_PPIE_FG01_P02: “Before my appointment, for the research, I was sent a very detailed list of all the precautions that were going to be taken, such as distancing, and PPE, you know, just everything. And it was really, for me really clearly, clearly written all in bullet points. So that, and plus the fact that I could just reply and say, oh, what about… And I’m really not sure about, and But yeah, I mean, there was so much reassurance before I went.”

Cardiovascular Disease _Trial participant_INT01: “As long as I know that where I am going has taken precautions and I’ve obviously you know, taking precautions as well, I’m not unduly worried about accessing doctors or hospitals.”

A barrier to attending hospital or clinical environments was not knowing what to expect with regards to Covid-19 regulations and rules at each site. PPIE contributors spoke of how the numerous Covid-19 rules and regulations often made them feel like *“naughty children”* [*Oral and Dental Health*_*PPIE_FG01_P02]* when they unknowingly or accidentally got something wrong. An additional barrier was not feeling safe under the new rules or regulations that had been put in place. PPIE contributors also felt that there were mixed messages regarding health care in the NHS; while some routine general care was suspended, certain trials were not, and they felt this sent mixed messages about whether it was safe to attend. They discussed that this could make people who are not participating in trials feel less valued. Participant engagement around safety messaging was key for trial staff; asking people what they wanted to know before coming in and what their concerns were helped staff to provide the right kind of information and reassurance. Trial staff found that it *“involved a lot more personal planning and a lot more reassurance than*, *than maybe you*, *you’d be used to for a trial*. *You know*, *as the co-ordinator I was speaking to my patients a lot more regularly*.” [*Oral and Dental Health*_*Trial staff member_INT03*].

Cancer _Trial participant_INT_01: “A few phone calls from my clinical trials nurse, and lots of paperwork, you know, instructing me how to conduct myself ….I always get sort of, you know, A4 sheets with, you know, you must do this, you must do that, etcetera, so, which is fine, yes. And I’ve always felt very safe. When I’ve gone. You know, I haven’t been anxious at all.”

Participant’s desire to know what to expect when visiting a clinical setting was reflective of their wish to be fully informed about what to expect when taking part in a clinical trial and how their contribution was going to be useful to the research overall. This went beyond the information often given on patient facing trial material and included wanting to know exactly what the process would be when they visited a clinical setting (irrespective of Covid-19). PPIE participants believed that videos showing the patient journey during a clinical visit would be really informative and help patients know what to expect. One primary care trial did provide a co-designed video with PPIE contributors to share what the process would be when they visited the clinical setting. The trial staff members discussed how this was implemented to *"try and welcome our patients in*, *so we did a little walk through*…*and the researcher just does a little bit of motivation talk at the beginning and then the nurses take them through what’ll happen on the day and who they might see” [Primary Care_Trial staff member_FG01_P3]*. Short videos explaining the research in lay terms often using animations or short clips of clinicians/researchers describing the research were seen to be an acceptable and useful tool in addition to participant information leaflets. It was recognised though that access to technology to watch a video could be a limiting factor for some patients, as such it was felt that these materials should be available to patients when first approached about the trial and shown in a clinical setting by one of the trial team.

Primary Care _PPIE_FG01_P01: “It has this lovely little YouTube video about you know what’s a good bug and what’s bad bug type of thing and it’s a lovely little cartoon make short and sharp but just brilliant you know. We are so visual nowadays, aren’t we in so many things and I just think that’s actually probably, you know, quite a hook to catch people, and then you go back to the sort of more boring or the thick patient information sheet and you know and the idea that somebody explains it alongside it, I think, is really, really helpful.”

Regardless of how informed they felt and the safety precautions put in place by the clinical team, a number of participants did not want to enter clinical settings. This was usually more clinically vulnerable or very unwell patients who felt that the benefits of taking part in the trial did not outweigh the potential negatives of exposure to Covid-19.

Cardiovascular Disease_PPIE_FG_02_P05: “I know that if I caught it, I would be very seriously ill with it. So I know, I will not attend these meetings. I have a friend who is waiting for a bypass. Three weeks, he’s in hospital. He had a patient moved from his board, because he had COVID. So it’s telling me that it’s still not a safe environment.”

Diabetes, Stroke and Respiratory Disorders_PPIE_FG01_PO3: “While that R number is high I know that I’m not comfortable with going into a healthcare setting and I’ll only go if it’s absolute essential. So somebody like me isn’t gonna sign up for a clinical trial, I don’t think, at the moment.”

## Discussion

This study identified three key facilitators to maintaining trial participation and PPIE engagement during the Covid-19 pandemic but also highly pertinent post Covid-19; 1) continued contact to facilitate trust, 2) a balanced move to remote methods of engagement, and 3) clear rules or information about what to expect when attending clinic visits to provide safety. These will be discussed considering the broader literature and how they can be used to facilitate trial setup and delivery in the post Covid-19 era.

### Building trust through communication

Continued contact through appropriate forms of communication was essential for keeping participants involved in and engaged with the trial. Our trial participants and PPIE contributors described how continued contact fostered trust in research staff which increased their willingness to remain in and return to research. This finding mirrors why people decide to take part in trials in the first place, as trust in healthcare professionals makes patients feel safe and secure when deciding to sign up for a trial [13]. The importance of continued contact between trial participants and research staff related to trust and maintaining relationships has been demonstrated in other studies. For example, Tashkandi et al [14] found that cancer patients’ preferred communication method during the Covid-19 pandemic was a telephone call, which the authors attributed to trust and reassurance in the established relationship with their oncologist. A cross-sectional survey found that 82% of patients (n = 6804) believed medical research should be conducted during epidemics [15] with trust in both the health professionals and government being cited as important for willingness to participate in research. Trust could be increased by prospectively informing patients of changes to research protocols following the Covid-19 pandemic, such as paused, remote, or delayed study visits [16].

Whilst maintaining continued contact was fundamental to trust and maintaining relationships, for staff it could increase workload and contribute to staff burnout; this had consequences for engaging research participants through the loss of staff and a lack of continuity of staff working on trials. There is a need for systems and processes that support efficient contact through adaptation of existing systems when new ways of working arise. Trial protocols are typically co-written with PPIE contributors, including a section for mitigating circumstances was considered prudent to help speed up agility for implementing changes acceptable to participants.

### A move to remote methods of engagement

Alternatives to clinical face-to-face appointments such as telephone, remote or home visits, were advantageous in some situations but were dependent on participants’ preferences and the reason for the appointment. Although these did come with some challenges such as concerns about the errors in diagnosis, loss of the human interaction and further pressures on staff workload.

Telemedicine may be advantageous in monitoring and providing care to people and could also support their participation in trials. For example, participants in an oncology trial were incentivised to attend imaging sessions during Covid-19 by the offer of a structured and timely virtual review of their images [17]. It is, however, also important to recognise concerns that were expressed by our participants about the potential for misdiagnosis during remote consultations and the inability to perform a proper physical examination. Several factors, including participant preference, will influence the suitability of remote visits. This is supported by a vignette-based study that asked participants their preferences for trial organisation and delivery [18]. Diverse preferences were found, emphasising that a one-size-fits all approach does not work; however, if trials are planned with consideration of participant preferences, the chance of participation may be increased by 30% [18]. Trial organisation should be tailored to an individual’s condition and personal characteristics, such as distance to the hospital, whether they are employed or disease severity.

Some participants reported that they may prefer to maintain remote visits rather than return to face-to-face appointments in the future showing how successful this move has been for certain types of appointments. Unexpected consequences of the pandemic such as these may result in long lasting trial modifications with positive effects. A survey of clinical research office personnel assessing perceptions, experiences and recommendations related to Covid-19 adjustments to trials found a perceived improvement in communication with participants, investigators and sponsors, patient safety, treatment efficacy, and the experience of both participants and staff [19]. Most respondents felt changes should continue post-pandemic including telehealth visits, remote consent and conducting diagnostic procedures or treatments off-site. Interestingly, the same study found clinicians and investigators were less positive about the changes, mainly those related to administering treatment and off-site procedures. This illustrates the need for careful evaluation of new ways of working from multiple perspectives and with consideration of all different outcomes. Other studies have found similar advantages and challenges to remote methods of engagement. Auchus and colleagues [20] found that while remote consultations may increase the likelihood of people attending appointments during a pandemic because they feel safer, there are challenges around technical issues, unfamiliarity, communication and a lack of human contact and connection. Boehm et al. [21] found that while there was a strong preference for video conferencing during the Covid-19 pandemic, a proportion would decline this due to technical limitations (17.3%) or because of the loss of personal contact with a physician (2.5%).

### Cost-benefit of participation

In our focus groups/interviews, trial participants who were vulnerable to Covid-19 felt there was value in taking part in clinical trials to gain access to drugs and treatment that they would not be offered. To date, this perspective on the risk-benefit of trial participation during pandemics from trial participants directly has been missed [22]. Willingness to join or continue with research appeared to be variable and dependant on the speciality area, disease severity and perception of Covid-19-related risks. Where the benefit of taking part in research was perceived to outweigh the risks, then there was a greater willingness to return. It must be recognised that although people who are at higher risk of infection could be more at-risk by participating in clinical trials during a pandemic, for certain areas like oncology, the potential benefits of taking part in a trial may outweigh the risks even where the pandemic makes individuals more vulnerable [23]. For these types of trials, the priority should be to devise risk reduction strategies to allow the trials to continue. Personalised risk-benefit analysis for research participation and concurrent risk minimisation is advocated, while still maintaining the trial integrity [24].

### Feeling fully informed

Feeling safe in the clinical setting was important to our participants and was experienced by the majority. Making sure participants were informed of what was expected of them in the clinical setting and the steps the site was taking to maintain social distancing and reduce the risk of Covid-19 transmission were key. Reassuringly, a study of older adults and caregivers during the Covid-19 pandemic found the vast majority (78%) felt safe or very safe attending a scheduled research appointment and that the medical centre was prepared/very prepared (82%) [22]. Deroose and colleagues [17] emphasised the importance of contacting participants prior to their visit to ask about possible symptoms to instil confidence that safety procedures are being followed. Trial staff may be able to reassure participants by sharing various safety measures such as body temperature checks, social distancing measures, hand sanitisation, surgical masks and one-way systems [25,26]. Participants in our study emphasised that having a designated point of contact may promote cohesive messages and create a trusted route for information sharing and for identifying and managing concerns, expectations and safety issues.

Our trial patient and PPIE participants had a strong desire to know as much as they could about the trial they were involved in and wanted to ensure that they were fully informed both of what would happen to them and what was expected of them. Our findings support previous literature that highlights that patients prefer reading more simplified information sheets without technical jargon [27].

The use of animations and videos has become more common in recent years and were viewed positively by our PPIE and trial patient participants. Research has suggested that the use of animations can improve participants knowledge about trials and their attitudes towards taking part [28]. Such approaches are also likely to be particularly effective for those with lower literacy skills, where English is not the first language, or those with a lower motivation to read lengthy, health-related information [29]. Short ‘elevator-pitch-style’ videos describing the importance of the research was welcomed by PPIE contributors.

### Strengths and limitations

An important strength of this research is the triangulation of the different perspectives of PPIE contributors, trial participants and trial staff members across a range of therapeutic areas and across sectors. PPIE contributors’ value came from providing a hypothetical and shared experience with others with the same condition, trial participants offered their own, immediate, lived experience, and trial staff gave insight into their experiences, how and why adaptations were made and reflected on the experience of staff and patients they worked with. During analysis we found good triangulation between all three groups; particularly between trial participants and PPIE contributors despite the differences in how they are involved in trials. Another strength is our inclusion of NIHR trials from across multiple specialities as this enabled us to explore differences in the impact of Covid-19, how these were mediated and how trial participants and PPIE contributors were impacted. This triangulation and coverage of trials across multiple specialities adds more depth to our understanding of engagement with trials during Covid-19.

It is important to note that we do not have equal representation from all participant groups/specialities. There was variability regarding how many participants in each group we were able to recruit, and some specialties were represented by larger participant numbers than others. However, as our aim was to capture experiences across multiple specialities and groups of participants and not undertake a direct comparison of differing experiences, we were less concerned about equal participant numbers across groups. To ensure a diverse mix of PPIE members and trial participants, we aimed for maximum variation based on age, gender and ethnicity. We recruited a diverse range of participants with regards to age and gender. However, despite our efforts to recruit those from ethnic minorities we were unable to achieve diversity with regards to ethnicity. Another potential limitation is the fact that the focus groups/interviews were undertaken by several different researchers. This could have impacted the consistency of the focus groups/interviews and the extent to which topics were probed. However, the topic guide followed a structured approach to the questions and topic areas which enabled consistency between interviewers. Also, there were no large differences between the questions and level of probing between different interviewers when the transcripts were checked before analysis. Lastly, all interviews and focus groups were undertaken online. This meant that those participating were those familiar with online videoing conferencing; this may have influenced their views on remote (video) consultations and may not be representative of the wider population, particularly those not familiar with online video conferencing.

### Implications for future practice

The findings from this research are highly pertinent to the continued commitment funders demand for efficient trial design and optimised delivery, whilst ensuring practice is informed by a participant-focused approach. The triangulation of views of PPIE contributors, trial participants and trial delivery staff are aligned with these principles and the ethos of the NIHR CRN that strives to uphold to the value of *‘no research about us*, *without us’* with a strong commitment to PPIE central to all its research. These principles are the ambition of international conducted research and thereby provides sensible recommendations and suggestions that are relevant to the conduct of trials in the UK and beyond.

This extensive qualitative enquiry provides pragmatic solutions for all clinical trials to consider of relevance across broad speciality therapeutic areas and settings. This should be considered at the design stage through to delivery to help maintain participant engagement with trials. Furthermore, these findings could help to future proof trials against future pandemics or other disruptions to planned delivery. The recommendations are focused on consideration of: 1) continued contact and communication to facilitate trust, 2) a balanced move to remote methods of engagement, and 3) clear rules or information about what to expect when attending clinic visits to provide safety. For example, by building in from the outset of trial design, the means for regular and sustained contact with initially PPIE contributors and then including trial participants during trial delivery will enhance opportunity for a patient-focused trial, cognate of what participants value and will help to build trust. This can be especially helpful if the key trial contact is trusted by the participants, easily reachable, and is a regular point of contact. Trial members should also contact individuals through their preferred means of communication (emails, letters, telephone etc) and where possible, tailor their appointments (clinic/home-visits/online/telephone) based on patient and preference and need. This should include providing up to date, clear information about steps taken to ensure safety in clinical settings as well more broadly what to expect in the clinical setting and what the visit will involve. Lastly, while our study identified that remote appointments are useful for monitoring and follow-up, it’s important to consider that not all patients prefer engaging remotely. Furthermore, remote PPIE meetings could make it harder to build rapport, small talk and getting to know one another. Therefore, providing a space for PPIE contributors to meet outside of the PPIE meetings can help foster group cohesion.

### Conclusion

In conclusion, this study is focused on overcoming the challenges of re-starting clinical trial research aligned with the NIHR Restart Framework [4] which is now superseded by the NIHR Reset and Recovery programme [30] (Fig 1). The findings are pertinent in a post-COVID-19 era and notably the three key facilitators for maintaining trial participation and PPIE engagement identified are relevant to the growth and delivery of the NIHR clinical trials portfolio: continued contact and communication to facilitate trust, a balanced move to remote methods of engagement, and clear information about what to expect when attending clinic visits to ensure patients are fully informed. Continued contact through appropriate forms of communication is crucial for keeping participants involved and engaged in trials, as it fosters trust in research staff, which increases their willingness to remain in and return to research. Remote methods of engagement, such as telephone, remote or home visits, were advantageous in some situations but came with some challenges such as concerns about the errors in diagnosis, loss of human interaction and further pressures on staff workload. The move to remote methods of engagement should be tailored to individual participant preferences, as a one-size-fits-all approach does not work. Feeling safe in the clinical setting was also important to our participants as well as making sure participants were informed of what was expected of them. Overall, these findings have implications for trial setup and delivery that go beyond the COVID-19 era. The strength has been the consideration of new ways of working from multiple perspectives–this study provides a unique amalgamation across PPIE contributor, trial participants and trial staff members across different clinical specialities and settings, providing a clear direction of how working in partnership can generate simple solutions to improve clinical trial efficiency and successful delivery.

## Supporting information

S1 File Supporting information. Topic Guides.
(PDF)

S2 File CORREQ checklist. (PDF)

S3 File Checklist. PLOSOne Clinical Studies Checklist.
(DOCX)

S4 File Appendix. DREC ethical approval.
(PDF)

S5 File Appendix. IRAS ethical approval.
(DOC)
