# Supplementary material for: Legacy lessons from the COVID-19 era to improve trial participation and retention: Views from trial participants, PPIE contributors and trial staff across the NIHR portfolio
Source: PLoS One. 2024 Feb 21;19(2):e0296343. doi: 10.1371/journal.pone.0296343 (PMC10880997; doi:10.1371/journal.pone.0296343)
Supplement: S1 File — Topic Guides. (PDF) [file pone.0296343.s001.pdf]

Subject: DREC ref: 250820/JC/307 - RESTART-COVID & US

Julie McDermott <J.K.McDermott@leeds.ac.uk>
to Julia Csikar, Jianhua Wu

Tue, 15 Sep

You are viewing an attached message. University of York Mail can't verify the authenticity of attached messages.

Dear Julia

DREC ref: 250820/JC/307

Study title: Public Engagement Programme to support the Restart of research studies after suspension due to COVID-19. (R
COVID & US)

Thank you for submitting the amended documents for the above application to the Dental Research Ethics Committee (DRE
documents have been reviewed and I am pleased to inform you that the application has been approved.

Documents reviewed

Document name	Version number and date
Ethics application	Dated 14/09/2020
Protocol	Version 1 25/08/2020
Participant information sheet	Version 2 14/09/2020
Consent form	Version 1 25/08/2020
Topic guide	Version 1 25/08/2020

With best wishes for the success of your project.

Please note: You are expected to keep a record of all your approved documentation, as well as documents such as s consent forms, signed consent forms, participant information sheets and all other documents relating to the study, risk assessments. This should be kept in your study file, and may be subject to an audit inspection. If your project is audited, you will be given at least 2 weeks' notice.

It is our policy to remind everyone that it is your responsibility to comply with Health and Safety, Data Protection an legal and/or professional guidelines there may be.

For and on behalf of

Dr Jianhua Wu

Deputy DREC Chair
