# Supplementary material for: Legacy lessons from the COVID-19 era to improve trial participation and retention: Views from trial participants, PPIE contributors and trial staff across the NIHR portfolio
Source: PLoS One. 2024 Feb 21;19(2):e0296343. doi: 10.1371/journal.pone.0296343 (PMC10880997; doi:10.1371/journal.pone.0296343)
Supplement: S5 File — IRAS ethical approval. (DOC) [file pone.0296343.s005.doc]

**
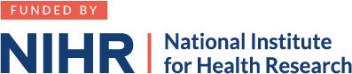

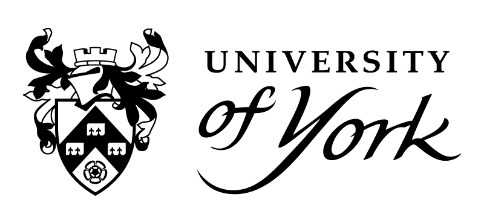

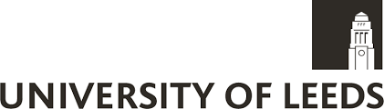
**

**PPIE**

**Focus Group/Interview topic guide (Version 2, 25/11/20)**

Objectives

We want to know your thoughts about the restarting of clinical research, and for some research projects returning to hospital for research visits, whilst we have safety measures in place for Covid-19.

Introduction

Aim: Introduction, review of the information sheet and ensure understanding

-Introduce self, researcher/dentist/other and affiliation -University of Leeds or other

-Purpose of the study, check that everyone has received and read the information sheet and invite any questions

-Ask if have seen, signed and returned consent form then read through the consent form as extra check – any qus at end

- tell everyone that you will be recording the session from now on

Background and setting the scene

Aim: To ensure we hear everyone’s names so that they can talk to each other using these in the discussion

-Go around the participants and ask them to confirm their name (as you will have to say their name as it appears on the screen to ask each one individually to speak) tell us if we’ve got your name correct and that’s how we can refer to you and if they would like to tell us where they are from (e.g. town)

**1.Question:** **What would you want to know about before a potential visit to hospital for a research visit?**

**OR**

**1B.Question:** (If more appropriate to your group)

**What would you want to know about before a potential visit to your home, school or care home by researcher/practitioners for a research visit?**

**Prompts for researcher of areas to direct discussion**:

**Opening**: Is anyone here enrolled on a research study currently? Or have been in the past? – how do you feel about it restarting up? What things you might have seen put in place that give you confidence about attending? OR Do you have any concerns?

OR (if no prior experience offered)

**Opening:** What if we talk a bit more generally about taking part - What do you think are the reasons behind why people take part in research? What do you think is the biggest motivator for people to take part? What are the benefits?

What do you think puts people off the most from taking part?

**Prompts for exploring decision- making:**

-What would you want to know in order to make your decision as to whether to take part? Information? Phone call? Option to delay?

-Who would you want to speak to?

-What would help you understand this information about risk and benefit? (follow up with some examples if required. eg. a discussion with your doctor, a written information leaflet, a video available on the internet or another format/combination of formats?)

-Who would you talk to about this?

-How important is it to you that the discussion is tailored to your personal circumstances?

-Do you think it would be influenced by healthcare workers/family/friends?

**Prompts for exploring weighing up the benefits and risks of taking part:**

-Do you think there would be any personal benefit to taking part?

-Do you think taking part would benefit wider society?

-Do you think it would disadvantage you in any way taking part?

-Do you think offering payments/incentives plays a role in people’s decision?

-Influenced by other health conditions you might have?

-Any influences from your personal background/beliefs that might put you off taking part?

**Other forms of delivery:**

How would you feel about the option to do it remotely/ at another setting? What benefits do you see to this? Do you have any concerns?

What information do you think is important to have? How receive it?

**Prompts on benefits or virtual delivery of research**

Convenience

Time saving

No travel or parking

Less risk

**Prompts on concerns over virtual delivery of research**

Lack of access to technology

Confidence to use technology

Language barriers

Sight or Hearing impairment

Confidence over care received

**Expectations of follow-up if any?**

**CONSIDER A SHORT COMFORT BREAK HERE (5-10 MINS)**

**2. Questions:** How would you feel about taking part in a research study trialing a vaccination for COVID-19?

**What would you want to know about a COVID-19 vaccine before taking part in a trial for it?**

What would help you to understand the risks and benefits of taking part? (follow up with some examples if required eg. a face-to-face discussion with your doctor, written information, an online resource with written information, pictures and videos).

**What’s your biggest concern about a COVID-19 vaccine?**

Prompts: Any concerns over safety? (Safe for humans? What are the ingredients? Research been too quick to be thorough? Worried that it is a Live vaccine? Side effects? Interaction with existing condition? Catching COVID-19? Where would you have to visit to get it and would there be a follow-up?) Distrust of research or researchers (particularly amongst ethnic minorities); Uncertainty (particularly in relation to trials; its links to randomisation) how much monitoring by physician you would receive

**What would be your biggest motivator to take part?** Low personal burden/impact or convenient research. General opinion on vaccination and how affects you? People’s opinion, those that are close/important to you would have an effect? Influence of physician, family or friends

Practitioners opinion/advice?

**Do you think there would be any personal benefit to taking part?** (including therapeutic benefits; closer monitoring; access to new treatments; gaining knowledge of own health)

**Do you think taking part would benefit public health/wider society?** make a difference and help? (Altruism -benefits to science; helping others)

**Do you think it might disadvantage you in any way?** (Fear and perceived risk (to health, of experimental treatment or adverse effects; to personal consequences, impact on condition/taking part in other research)

**What do you think would be the biggest barrier to you taking part?** Practical difficulties (including additional procedures or appointments; transport; costs; work or caring responsibilities),Confidence or trust in the physician or the research; Treatment preferences (for specific therapy; against placebo, desire for choice and concerns over randomisation), Depends on current health status and fluctuations in your condition

**To bring in at any point it seems appropriate: How would you feel about wearing a see-through face mask?** i.e. so people can see your mouth when you speak. What would you think about other people wearing them?

Interview closure

-Anything else you would like to mention/Any areas that you think we should have covered?

-Any questions?

-Thanks and how to contact us further if you should want to

**
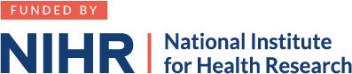

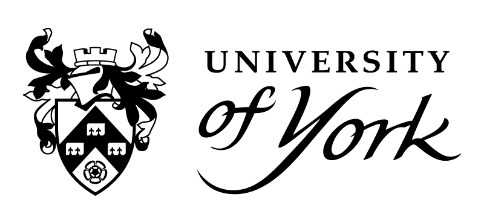

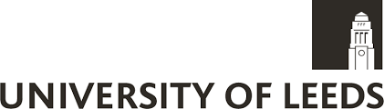
**

**Trial Staff Members**

**Focus Group/Interview topic guide (Version 1, 05/01/21)**

Objectives

We want to know of any changes brought in or not and your thoughts about the restarting of clinical research, and for some research projects returning to hospital for research visits, whilst we have safety measures in place for Covid-19. To identify barriers overcome and facilitators to identify best practice

Introduction

Aim: Introduction, review of the information sheet and ensure understanding

-Introduce self, researcher/dentist/other and affiliation -University of Leeds or other

-Purpose of the study, check that everyone has received and read the information sheet and invite any questions

-Ask if have seen, signed and returned consent form then read through the consent form as extra check – any qus at end

- All participants are asked that the focus group is to be treated with confidence and not reveal outside of it what others have said or to identify them

- tell everyone that you will be recording the session from now on

Breaking the ice and setting the scene

Aim: To ensure we hear everyone’s names so that they can talk to each other using these in the discussion

-Go around the participants and ask them to confirm their name (as you will have to say their name as it appears on the screen to ask each one individually to speak) tell us if we’ve got your name correct and that’s how we can refer to you and if they would like to tell us where they are from (e.g. town)

**1.Question:** to start would you be able to give us a brief overview about what has happened to your trails since March 2020? Across other sites/trusts?

Paused? Difficulties? Re-started?

Has it relied upon pharmacy? Radiography? Others?

What makes a site able to open earlier?

Patients noticed changes? Re-consent – especially important for opt-out (schools), patients not wanting to take part during COVID, balancing ethics vs getting people into trial

What about the people delivering – have you made any protocol amendments? What did they include?

Have been easier for opening trial or patient benefit?

Ideas:

Short videos – keeping safe, no public transport, one-way system, hands, sanitizer stations, partners not allowed unless carers?– preparing people what to expect and managing expectations.

Any service evaluation taken place- surveying patients when they come in- what patients want to see and how to find it, surveying sites

Home phlebotomy – shielded people option

Hoe triage and symptoms/COVID-19 test

Timeline flexibility – for deliverables on the trial to get people through eg MRI scanning

Pharmacy and Radiology input – prioritized over other COVID-19 stuff

Prioritization during waves and tiers and at different trusts. No central sign-off? Site dependent? Priority of restart?

From PPI – able to share trusted sources for more information

How patients feel about modifications- such as less MRI scanning?

Can people only attend on their own and have you made any modifications because of this?

Schools

Prioritization research vs school activities? Normality?

Re-consent – especially important for opt-out (schools)

Restricting people coming in vs business as usual? Zoom problematic with whole class, no visual if on phone, used chat function?

Interview closure

-Anything else you would like to mention/Any areas that you think we should have covered?

-Any questions?

-Thanks and how to contact us further if you should want to

**
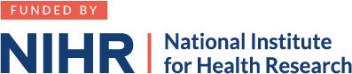

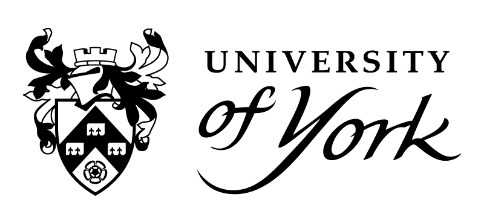

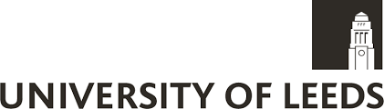
**

**Trial participants.**

**Focus Group/Interview topic guide (Version 2, 25/11/20)**

Objectives

We want to know your thoughts about the restarting of clinical research, and for some research projects returning to hospital for research visits, whilst we have safety measures in place for Covid-19.

Introduction

Aim: Introduction, review of the information sheet and ensure understanding

-Introduce self, researcher/dentist/other and affiliation -University of Leeds or other

-Purpose of the study, check that everyone has received and read the information sheet and invite any questions

-Ask if have seen, signed and returned consent form then read through the consent form as extra check – any qus at end

- tell everyone that you will be recording the session from now on

Background and setting the scene

Aim: To ensure we hear everyone’s names so that they can talk to each other using these in the discussion

-Go around the participants and ask them to confirm their name (as you will have to say their name as it appears on the screen to ask each one individually to speak) tell us if we’ve got your name correct and that’s how we can refer to you and if they would like to tell us where they are from (e.g. town)

**1.Question:** **What would you want to know about before a potential visit to hospital for a research visit?**

**OR**

**1B.Question:** (If more appropriate to your group)

**What would you want to know about before a potential visit to your home, school or care home by researcher/practitioners for a research visit?**

**Prompts for researcher of areas to direct discussion**:

**Opening**: Is anyone here enrolled on a research study currently? Or have been in the past? – how do you feel about it restarting up? What things you might have seen put in place that give you confidence about attending? OR Do you have any concerns?

OR (if no prior experience offered)

**Opening:** What if we talk a bit more generally about taking part - What do you think are the reasons behind why people take part in research? What do you think is the biggest motivator for people to take part? What are the benefits?

What do you think puts people off the most from taking part?

**Prompts for exploring decision- making:**

-What would you want to know in order to make your decision as to whether to take part? Information? Phone call? Option to delay?

-Who would you want to speak to?

-What would help you understand this information about risk and benefit? (follow up with some examples if required. eg. a discussion with your doctor, a written information leaflet, a video available on the internet or another format/combination of formats?)

-Who would you talk to about this?

-How important is it to you that the discussion is tailored to your personal circumstances?

-Do you think it would be influenced by healthcare workers/family/friends?

**Prompts for exploring weighing up the benefits and risks of taking part:**

-Do you think there would be any personal benefit to taking part?

-Do you think taking part would benefit wider society?

-Do you think it would disadvantage you in any way taking part?

-Do you think offering payments/incentives plays a role in people’s decision?

-Influenced by other health conditions you might have?

-Any influences from your personal background/beliefs that might put you off taking part?

**Other forms of delivery:**

How would you feel about the option to do it remotely/ at another setting? What benefits do you see to this? Do you have any concerns?

What information do you think is important to have? How receive it?

**Prompts on benefits or virtual delivery of research**

Convenience

Time saving

No travel or parking

Less risk

**Prompts on concerns over virtual delivery of research**

Lack of access to technology

Confidence to use technology

Language barriers

Sight or Hearing impairment

Confidence over care received

**Expectations of follow-up if any?**

Are you more aware of research because of COVID research?

**CONSIDER A SHORT COMFORT BREAK HERE (5-10 MINS)**

**2. Questions:** How would you feel about taking part in a research study trialing a vaccination for COVID-19?

**What would you want to know about a COVID-19 vaccine before taking part in a trial for it?**

What would help you to understand the risks and benefits of taking part? (follow up with some examples if required eg. a face-to-face discussion with your doctor, written information, an online resource with written information, pictures and videos).

**What’s your biggest concern about a COVID-19 vaccine?**

Prompts: Any concerns over safety? (Safe for humans? What are the ingredients? Research been too quick to be thorough? Worried that it is a Live vaccine? Side effects? Interaction with existing condition? Catching COVID-19? Where would you have to visit to get it and would there be a follow-up?) Distrust of research or researchers (particularly amongst ethnic minorities); Uncertainty (particularly in relation to trials; its links to randomisation) how much monitoring by physician you would receive

**What would be your biggest motivator to take part?** Low personal burden/impact or convenient research. General opinion on vaccination and how affects you? People’s opinion, those that are close/important to you would have an effect? Influence of physician, family or friends

Practitioners opinion/advice?

**Do you think there would be any personal benefit to taking part?** (including therapeutic benefits; closer monitoring; access to new treatments; gaining knowledge of own health)

**Do you think taking part would benefit public health/wider society?** make a difference and help? (Altruism -benefits to science; helping others)

**Do you think it might disadvantage you in any way?** (Fear and perceived risk (to health, of experimental treatment or adverse effects; to personal consequences, impact on condition/taking part in other research)

**What do you think would be the biggest barrier to you taking part?** Practical difficulties (including additional procedures or appointments; transport; costs; work or caring responsibilities),Confidence or trust in the physician or the research; Treatment preferences (for specific therapy; against placebo, desire for choice and concerns over randomisation), Depends on current health status and fluctuations in your condition

**To bring in at any point it seems appropriate: How would you feel about wearing a see-through face mask?** i.e. so people can see your mouth when you speak. What would you think about other people wearing them?

Interview closure

-Anything else you would like to mention/Any areas that you think we should have covered?

-Any questions?

-Thanks and how to contact us further if you should want to
