# Supplementary material for: Legacy lessons from the COVID-19 era to improve trial participation and retention: Views from trial participants, PPIE contributors and trial staff across the NIHR portfolio
Source: PLoS One. 2024 Feb 21;19(2):e0296343. doi: 10.1371/journal.pone.0296343 (PMC10880997; doi:10.1371/journal.pone.0296343)
Supplement: S2 File — (PDF) [file pone.0296343.s002.pdf]

Professor Susan Pavitt
Director of DenTCRU [Dental Translational and Clinical Research Unit] part of the NIHR Leeds Clinical Research Facility
University of Leeds Faculty of Medicine and Health
The Faculty of Medicine and Health, University of Leeds
Clarendon Way
Leeds
LS2 9LU

Email: approvals@hra.nhs.uk

21 December 2020

Dear Professor Pavitt

HRA and Health and Care Research Wales (HCRW) Approval Letter

**Study title:**	**Restarting clinical research after suspension due to COVID-19: RESTART-COVID & US**
**IRAS project ID:**	**288766**
**REC reference:**	**20/PR/0633**
**Sponsor**	**University of Leeds**

I am pleased to confirm that **HRA and Health and Care Research Wales (HCRW) Approval** has been given for the above referenced study, on the basis described in the application form, protocol, supporting documentation and any clarifications received. You should not expect to receive anything further relating to this application.

Please now work with participating NHS organisations to confirm capacity and capability, in line with the instructions provided in the "Information to support study set up" section towards the end of this letter.

How should I work with participating NHS/HSC organisations in Northern Ireland and Scotland?
HRA and HCRW Approval does not apply to NHS/HSC organisations within Northern Ireland and Scotland.

If you indicated in your IRAS form that you do have participating organisations in either of these devolved administrations, the final document set and the study wide governance report

(including this letter) have been sent to the coordinating centre of each participating nation. The relevant national coordinating function/s will contact you as appropriate.

Please see IRAS Help for information on working with NHS/HSC organisations in Northern Ireland and Scotland.

How should I work with participating non-NHS organisations?
HRA and HCRW Approval does not apply to non-NHS organisations. You should work with your non-NHS organisations to obtain local agreement in accordance with their procedures.

What are my notification responsibilities during the study?

The standard conditions document "*After Ethical Review – guidance for sponsors and investigators*", issued with your REC favourable opinion, gives detailed guidance on reporting expectations for studies, including:

- Registration of research
- Notifying amendments
- Notifying the end of the study

The HRA website also provides guidance on these topics, and is updated in the light of changes in reporting expectations or procedures.

Who should I contact for further information?
Please do not hesitate to contact me for assistance with this application. My contact details are below.

Your IRAS project ID is **288766**. Please quote this on all correspondence.

Yours sincerely,
Juliana Araujo

Approvals Specialist

Email: approvals@hra.nhs.uk

Copy to: *Mrs Jean Uniacke*

List of Documents

The final document set assessed and approved by HRA and HCRW Approval is listed below.

Document	*Version*	*Date*
Evidence of Sponsor insurance or indemnity (non NHS Sponsors only) [Public and Employers Indemnity]	1	26 August 2020
HRA Schedule of Events [dated]	1	14 October 2020
Interview schedules or topic guides for participants [Focus Group/Interview Topic Guide - tracked and clean]	2	25 November 2020
IRAS Application Form [IRAS_Form_14102020]		14 October 2020
IRAS Checklist XML [Checklist_14102020]		14 October 2020
Letter from funder [Award Letter]	1	05 August 2020
Letter from sponsor [Letter from Sponsor]	1	13 October 2020
Letters of invitation to participant [Letter of invitation to participate]	1	09 September 2020
Letters of invitation to participant [Letter of invitation to participate - gatekeepers]	1	09 September 2020
Non-validated questionnaire [Participant Demographics Form - tracked and clean]	2	12 November 2020
Organisation Information Document [Organisation Information Document]	1	07 October 2020
Other [Final Letter - Response to Ethical Review Favourable Opinon 8th December 2020]		
Other [RESTART Confidentiality agreement for transcriber v1 09.09.2020]	1.0	09 September 2020
Participant consent form [Consent Form - tracked and clean]	2	30 November 2020
Participant information sheet (PIS) [Patient Information sheet]	1	06 October 2020
Participant information sheet (PIS) [Patient Information sheet gatekeepers]	1	12 October 2020
Research protocol or project proposal [Protocol]	1	09 September 2020
Response to Additional Conditions Met		
Summary CV for Chief Investigator (CI) [IRAS 2020 Short CV PAVITT]	1.0	01 April 2020
Summary of any applicable exclusions to sponsor insurance (non-NHS sponsors only) [Professional Indemnity Proof of Cover]	1	01 October 2020
20PR0633 IRAS 288766 Favourable with Conditions - 9.11.20.pdf		09 November 2020
20PR0633 IRAS 288766 Favourable with Conditions - 9.11.20.rtf		09 November 2020
288766, 20/PR/0633, SE05 HRA Approval PRS email confirmation template.eml		14 October 2020
288766, 20/PR/0633, SE11 – Application Valid – PRS REC review - non-commercial.eml		16 October 2020
288766, 20/PR/0633, SE30 Status Update - Favourable Opinion.eml		09 November 2020
IRAS_288766_Acknowledgement_for_documentation_received_following_a_FO_FIFO_AC_21_12_2020.rtf		21 December 2020

IRAS project ID	288766

Information to support study set up

The below provides all parties with information to support the arranging and confirming of capacity and capability with participating NHS organisations in England and Wales. This is intended to be an accurate reflection of the study at the time of issue of this letter.

Types of participating NHS organisation	Expectations related to confirmation of capacity and capability	Agreement to be used	Funding arrangements	Oversight expectations	HR Good Practice Resource Pack expectations
This study involves NHS Participant Identification Centres (PICs)	PIC activities should not commence until a PIC Agreement is in place. HRA and HCRW recommend use of the standard Participating NHS Organisation to PIC agreement available here.	HRA and HCRW recommend use of the standard Participating NHS Organisation to PIC agreement, available here.	External funding has been secured by Y&H NIHR CRN. No funding is available to NHS sites.	A local collaborator is expected to be in place at the NHS site to oversee access of the external research team to the NHS premises.<br>The Chief Investigator is expected to be able to answer any study queries from the PIC sites.	Researchers at the NHS PIC sites are expected to have employment contracts already in place, so letters of access are not required. As it is anticipated that all patients are contacted by telephone, zoom or post, no pre-engagement checks or occupational health clearance are required.<br><br>Additionally, as this study is taking place in GP practices you are advised to contact the primary care management function to follow local processes.

Other information to aid study set-up and delivery

This details any other information that may be helpful to sponsors and participating NHS organisations in England and Wales in study set-up.
All participant invitation documentation will be provided to be sent out and participants will be requested to contact the researchers directly about the study and taking part
